# Sorting nexin-21 is a scaffold for the endosomal recruitment of huntingtin

**DOI:** 10.1242/jcs.211672

**Published:** 2018-09-10

**Authors:** Chris M. Danson, Neil Pearson, Kate J. Heesom, Peter J. Cullen

**Affiliations:** 1 School of Biochemistry, Biomedical Sciences Building, University of Bristol, Bristol BS8 1TD, UK; 2 Proteomics Facility, School of Biochemistry, Biomedical Sciences Building, University of Bristol, Bristol BS8 1TD, UK

**Keywords:** Huntingtin, Retromer, Septin, Sorting nexin

## Abstract

The endo-lysosomal network serves an essential role in determining the fate of endocytosed transmembrane proteins and their associated proteins and lipids. Sorting nexins (SNXs) play a central role in the functional organisation of this network. Comprising over 30 proteins in humans, SNXs are classified into sub-groups based on the presence of additional functional domains. Sorting nexin-20 (SNX20) and sorting nexin-21 (SNX21) comprise the SNX-PXB proteins. The presence of a predicted protein-protein interaction domain, termed the PX-associated B (PXB) domain, has led to the proposal that they function as endosome-associated scaffolds. Here, we used unbiased quantitative proteomics to define the SNX21 interactome. We reveal that the N-terminal extension of SNX21 interacts with huntingtin (Htt) whereas the PXB domain appears to associate with septins, a family of cytoskeletal- and membrane-associated proteins. In establishing that these interactions are sufficient for SNX21 to recruit Htt and septins on to an endosomal population, we reveal a scaffolding function for this sorting nexin. Our work paves the way for a more-detailed mechanistic analysis of the role(s) of the SNX-PXB proteins in endosomal biology.

## Abstract (precis)

**Summary:** A potential scaffolding function for SNX21 paves the way for a more-detailed mechanistic analysis of the role(s) of this protein in endosomal biology.

## INTRODUCTION

The human genome is estimated to encode in excess of 5000 transmembrane proteins (Fagerberg et al., 2010; Babcock and Li, 2014). It is essential that these proteins are efficiently sorted and transported to their correct subcellular location in order to establish and maintain the function of numerous membrane-bound organelles. Equally, for cells, tissues and organisms to adapt to a changing environment, these sorting and transport events must be controlled, to allowing re-modelling of the transmembrane content and hence functionality of the specified organelle. One series of inter-connected membranous compartments that serves an essential and evolutionarily conserved role in the sorting and transport of numerous transmembrane proteins is the endosomal network (Huotari and Helenius, 2011; Burd and Cullen, 2014; Klumperman and Raposo, 2014).

Classically, the endosomal network receives transmembrane proteins and their associated proteins and lipids, together termed cargos, primarily from two sources: the plasma membrane via the process of endocytosis, and the biosynthetic pathway through direct transport from the *trans*-Golgi network (TGN) (Maxfield and McGraw, 2004; Johannes and Popoff, 2008; Grant and Donaldson, 2009; Huotari and Helenius, 2011; Johannes and Wunder, 2011; Hsu et al., 2012). These pathways converge at the sorting endosome, where sorting events are initiated that determine the fate of individual cargos. Cargos can be sorted into forming intraluminal vesicles (ILVs) that bud from the limiting membrane of the endosome (Henne et al., 2011; Schöneberg et al., 2016). Through iterative rounds of sorting and ILV biogenesis coupled with the maturation of the endosome into a compartment that becomes competent to fuse with the lysosome, those ILV-associated cargos undergo lysosomal-mediated degradation (Kummel and Ungermann, 2014). Alternatively, cargo can be retrieved from this destructive fate before being transported to the plasma membrane (Maxfield and McGraw, 2004; Hsu et al., 2012; Burd and Cullen, 2014), to the biosynthetic pathway (Johannes and Wunder, 2011; Burd and Cullen, 2014) or to a variety of other destinations that include lysosome-related organelles and elements of the autophagic pathway (Luzio et al., 2014; Carlsson and Simonsen, 2015). Underpinning the importance of the network is an increasing body of evidence suggesting that perturbed endosomal sorting and transport is a contributing factor in various human diseases, most notably neurodegenerative diseases (Schreij et al., 2016).

Sorting nexins (Kurten et al., 1996) are an ancient evolutionarily conserved family of proteins that play a central role in the functional organisation of the endosomal network (Carlton et al., 2004; Cullen, 2008; Cullen and Korswagen, 2012; Teasdale and Collins, 2012; Chi et al., 2015; van Weering et al., 2010; Gallon and Cullen, 2015). Comprising over 30 proteins in humans, the defining feature of all sorting nexins is the presence of a phox homology (PX) domain (Teasdale and Collins, 2012). This modular domain typically functions to bind phosphoinositides, most commonly the early endosome-enriched phosphatidylinositol 3-monophosphate [PtdIns(3)P] (Cheever et al., 2001; Ellson et al., 2001; Xu et al., 2001; Kanai et al., 2001) – an interaction that serves to aid the targeting of the peripheral sorting nexins to the cytosolic surface of endosomes (Teasdale et al., 2001; Cozier et al., 2002; Stockinger et al., 2002; Zhong et al., 2002). Sorting nexins are further classified into sub-groups based on the presence of additional functional domains, that including BAR, PDZ, FERM-like, RGS and SH3 domains (Florian et al., 2001; Worby et al., 2001; Zheng et al., 2001; Burden et al., 2004; Carlton et al., 2004; Joubert et al., 2004; Wassmer et al., 2009; van Weering et al., 2012b).

One SNX sub-group that in humans comprises sorting nexin-20 (SNX20) and sorting nexin-21 (SNX21) are the SNX-PXB proteins (Zeng et al., 2002; Schaff et al., 2008; Teasdale and Collins, 2012). These proteins possess a C-terminal PX-associated B (PXB) domain which displays a tetratricopeptide repeat (TPR) fold that is typically involved in protein-protein interactions (Clairfeuille et al., 2015). While SNX20 and SNX21 share the same overall domain organisation and approximately 40% sequence conservation at the amino acid level, they are distinguished by an N-terminal extension in SNX21 that is predicted to include a short α-helical structure (Clairfeuille et al., 2015). Both proteins conform to the classical view of sorting nexins, as they associate with the endosomal network through the interaction of their respective PX domains with endosome-enriched phosphoinositides (Clairfeuille et al., 2015). The prevailing view, therefore, is that the SNX-PXB proteins serve a role as endosome-associated scaffolds (Clairfeuille et al., 2015).

That being said, little is known of the functional role of SNX-PXB proteins. One study has proposed a role for SNX20 in the endosomal trafficking of the integral adhesion receptor for P-selectin glycoprotein ligand 1 (PSGL-1, also known as SELPLG), although an SNX20-knockout mouse displayed no phenotype relating to PSGL-1 function in endothelial cell adhesion (Schaff et al., 2008). In addition, the ability of SNX20 to directly associate with the cytosolic domain of PSGL-1 has recently been questioned (Clairfeuille et al., 2015). In an RNAi loss-of-function screen, suppression of SNX21 expression had a minor effect on the degradative sorting of internalised EGF receptor, although the mechanistic basis of this modest effect remains unclear (Danson et al., 2013). Finally, the *SNX20* gene locus has recently been linked to inflammatory bowel disease, in particular Crohn's disease, through genome-wide association in African Americans (Brant et al., 2016).

In the present study we have used unbiased quantitative proteomics to define those proteins that associate with SNX21, revealing that the SNX21 N-terminal extension interacts with the Huntington's disease protein huntingtin (Htt) (Saudou and Humbert, 2016) whereas the PXB domain appears to associate with various members of the septin family of cytoskeletal- and membrane-associated proteins (Mostowy and Cossart, 2012): an interaction that is also observed in SNX20. In establishing that these interactions are sufficient for SNX21 to recruit Htt and septins on to an endosomal population, we reveal a scaffolding function for this sorting nexin. Our work paves the way for a more detailed mechanistic analysis of the role(s) of the SNX-PXB proteins in endosomal biology.

## RESULTS

### SNX21 is associated with the endocytic network

**Fig. 1.**
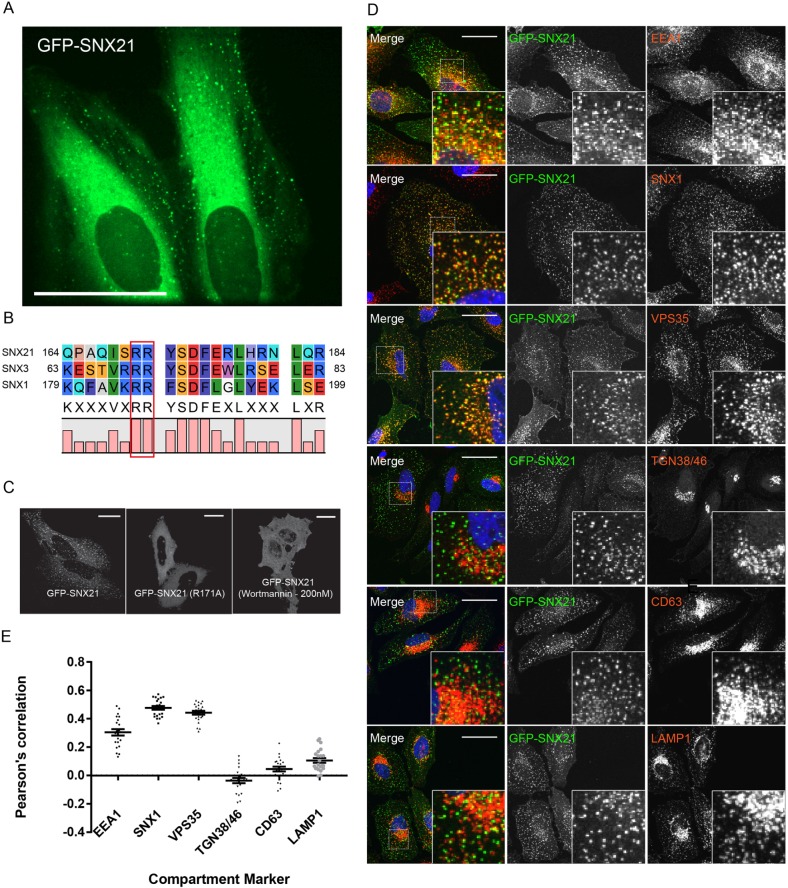
**The PX domain of SNX21 is required for its targeting to highly dynamic PtdIns(3)P-enriched early endosomes.** (A) HeLa cells stably expressing a plasmid encoding eGFP-SNX21 were imaged live. A selected frame of a live movie depicting the localisation of GFP-SNX21 to highly dynamic, peripherally localised punctae. Scale bar: 40 µm. (B) Protein sequence alignments between SNX1, SNX3 and SNX21 reveal the conserved arginine residues in the PX domain of SNX21 implicated in the binding to phosphoinositides. (C) HeLa cells were transfected with DNA encoding eGFP-SNX21, fixed and imaged: wild-type SNX21 localises to peripheral punctae, SNX21 R171A is cytosolic, as is wild-type SNX21 upon inactivation of PI3-kinase via treatment with wortmannin (200 nM). Scale bars: 20 µm. (D) HeLa cells were virally transduced to express GFP-SNX21 and co-immunostained for endogenous proteins representative of various trafficking compartments and imaged using confocal microscopy. Scale bars: 20 µm. (E) Quantitative colocalisation analysis between GFP-SNX21 and endogenous compartment markers. Graph represents the mean of >22 cells quantified; error bars show s.e.m.

The available structural data are consistent with a potential scaffolding role for SNX21 (Clairfeuille et al., 2015). For other sorting nexins with scaffolding roles, we used stable isotope labelling of amino acids in cell culture (SILAC)-based quantitative proteomics coupled with high-affinity GFP-nanotrap immunoisolation of GFP fusion proteins to reveal functionally relevant protein-protein interactions (Steinberg et al., 2013; McGough et al., 2014a,b; McMillan et al., 2016; McNally et al., 2017; Simonetti et al., 2017). As a prelude to applying this methodology to SNX21, we first isolated a cDNA encoding full-length human SNX21. This was cloned into a lentiviral vector to encode an N-terminal GFP-tagged SNX21 chimera (GFP-SNX21). Titration of the resultant lentivirus generated a population of HeLa cells displaying high levels of transduction in which GFP-SNX21 was associated with dispersed and dynamic cytosolic puncta (Fig. 1A). Recently, Clairfeuille and colleagues established that SNX21 is recruited to early endosomes through the binding of its PX domain to phosphatidylinositol 3-monophosphate [PtdIns(3)P] and phosphatidylinositol 4,5-bisphosphate [PtdIns(4,5)P_2_] (Clairfeuille et al., 2015). To validate our GFP-SNX21 chimera, we therefore first introduced a mutation into a conserved arginine residue within the SNX21 PX domain, arginine 171, the equivalent residue of which is essential for phosphoinositide binding to the PX domain of other sorting nexins (Fig. 1B) (Teasdale and Collins, 2012). The resultant SNX21(R171A) mutant failed to localise to cytosolic punctae and instead was distributed throughout the cytosol (Fig. 1C). A similar loss of punctate association was observed with wild-type SNX21 following incubation with the PI3-kinase inhibitor wortmannin (Fig. 1C).

**Fig. 2.**
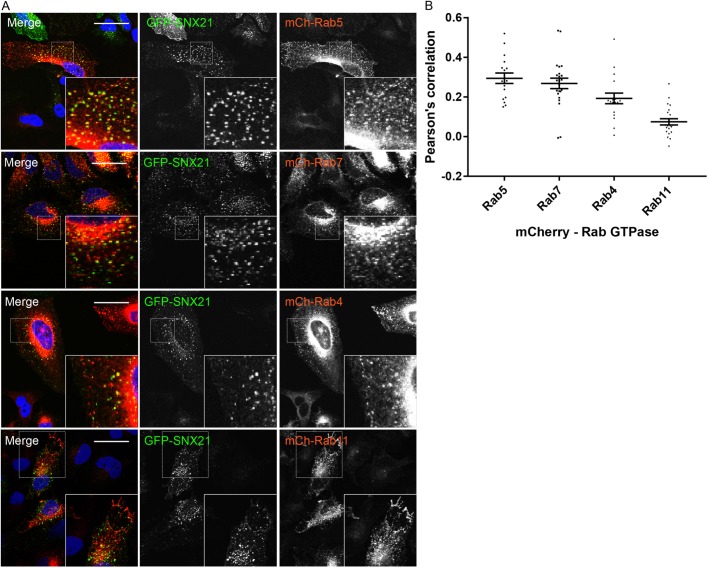
**GFP-SNX21 is highly enriched on early elements of the maturing endo-lysosomal network.** (A) HeLa cells were virally transduced to express GFP-SNX21 and various mCherry-Rabs that associate with temporally and spatially segregated sorting compartments, prior to fixation and imaging using confocal microscopy. Scale bars: 20 µm. (B) Quantitative colocalisation analysis between GFP-SNX21 and mCherry-Rab markers using Volocity. Graph represents the mean of >15 cells quantified from each. Error bars show s.e.m.

Next, we performed a series of confocal imaging experiments where we co-stained GFP-SNX21 expressing HeLa cells with standard markers for early endosomes (EEA1), early-to-late transition endosomes (SNX1, VPS35), late endosomes (LAMP1, CD63) and the *trans*-Golgi network (TGN38) (Fig. 1D). Quantification of the resultant images through Pearson co-localisation and correlation coefficients confirmed that the GFP-SNX21-labelled punctae were predominantly early endocytic in character, extending from early endosomes through to early-to-late transition endosomes (Fig. 1E; Table S4). We extended this analysis through a similar co-localisation analysis with mCherry-tagged versions of Rab4, Rab5, Rab7 and Rab11, again quantifying images by means of Pearson analysis (Fig. 2A; Table S5). Co-localisation was observed with Rab5-labelled early endosomes and Rab7-labelled late endosomes, as well as elements of the Rab4/Rab11 recycling endosomes (Fig. 2B). Together, these data confirm and extend the characterisation of the association of SNX21 with early elements of the maturing endo-lysosomal network (Clairfeuille et al., 2015), and establish that the N-terminal tagging of SNX21 with GFP does not adversely affect its ability to associate with, and localise to, these endocytic compartments.

### Quantitative proteomics-based identification of the SNX21 interactome

**Fig. 3.**
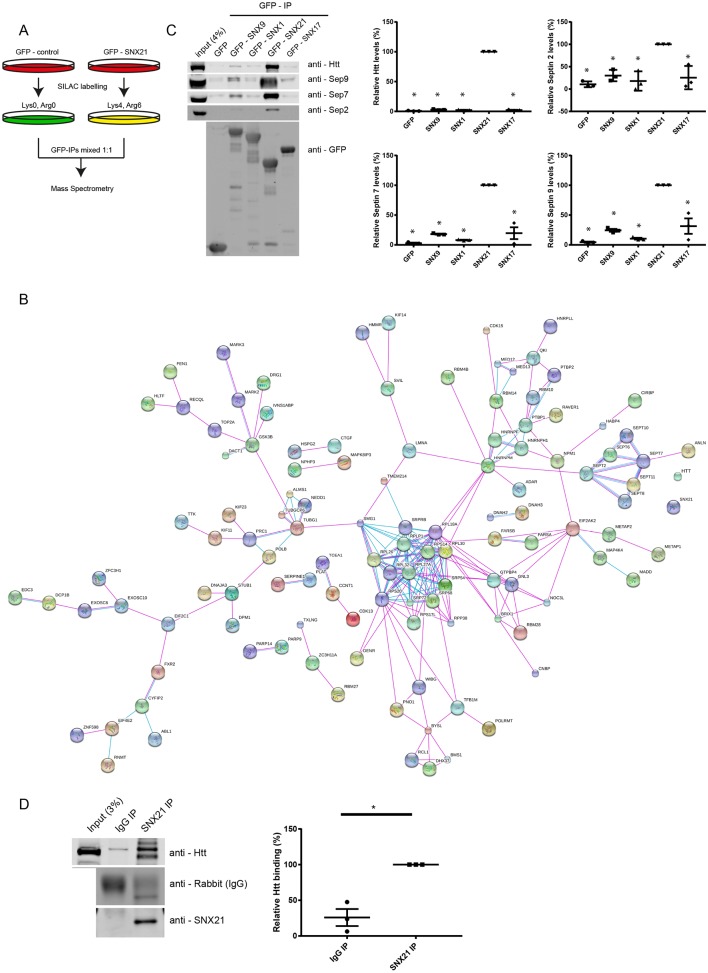
**SILAC and western blot analyses of the SNX21 interactome reveals interactions between SNX21, huntingtin and septins.** (A) RPE1 cells were virally transduced to express GFP-SNX21 or GFP; the GFP-expressing cells were cultured in normal (R0K0) medium, whereas the GFP-SNX21-expressing cells were grown in ‘medium’-isotope labelled medium (R6K4), prior to lysis and GFP-nanotrap-mediated precipitation of the GFP tags. Precipitates were pooled, separated by SDS-PAGE and subjected to gel walking LC-MS/MS analysis on an ORBITRAP mass spectrometer. (B) Using the criteria referred to in the Materials and Methods, we used an algorithm (https://string-db.org/) to search for functional groupings within the SNX21 interactome. Both lines represent known interactions from curated databases (pink) and those that have been experimentally determined (purple). The dashed lines represent previously published experimental links between proteins, which have not been detected via STRING analysis. Larger nodes represent proteins of known or predicted 3D structure. (C) Representative western analysis of HEK293-T cells transiently transfected to express GFP-tagged SNXs and the resultant immunoprecipitates from the corresponding GFP-nanotrap isolates probed with antibodies against the respective endogenous proteins. Quantification of western analysis confirming the specific nature of these interactions with respects to SNX21. Graphs represent the mean of three biological replicates with error bars indicating s.e.m. Values for the amount of co-immunoprecipitated protein with respective GFP-SNXs were normalised relative to that of the GFP tag alone and two-tailed unpaired *t*-tests were used to compare the significance of change with respect to Htt or septin binding between SNX21 and the other SNXs analysed (*P*<0.05). (D) Representative western analysis of endogenous SNX21 and rabbit IgG co-immunoprecipitation of huntingtin. Graph represents the mean of three biological replicates with error bars indicating s.e.m. Within each biological repeat, Htt binding to SNX21 was set at 100% and values for binding to IgG adjusted accordingly. Significance of change (*P<0.05) was determined using unpaired two-tailed *t*-tests and values for precipitated Htt were normalised relative to the intensity of the respective IgG bands.

To perform SILAC-based quantitative proteomics, we cultured GFP- and GFP-SNX21-expressing RPE-1 cells in light (R0K0) and medium (R6K4) media for at least six doublings to ensure steady-state protein labelling. Immuno-isolation of the GFP tag was achieved through GFP-nanotrap prior to mixing of the two samples and protein resolution on SDS-PAGE and protein identification by LC-MS/MS (Fig. 3A). The resultant data (Table S2) were filtered first by exclusion of those proteins identified through a single peptide and secondly, the exclusion of proteins that showed a quantified enrichment ratio of GFP-SNX21:GFP of less than 20. This resulted in a list of 287 proteins (Table S3), of which the top two hits were Huntingtin (Htt) and sacsin, proteins that when mutated lead to the neurodegenerative diseases Huntington's disease (HD) and autosomal-recessive spastic ataxia of Charlevoix-Saguenay (ARSACS), respectively (Saudou and Humbert, 2016). A string analysis of the top hits (defined by those proteins 100-fold enriched and with a protein score of >50) identified a network of experimentally defined associations within the interactome (Fig. 3B). Most notable was the presence of SEPT2, SEPT6, SEPT7, SEPT8, SEPT10 and SEPT11, all members of the septin family of filamentous heteromeric GTPases (Mostowy and Cossart, 2012). Septins are associated with the cytoskeleton and with cellular membranes, and have emerging roles in a number of membrane-based processes, including poorly defined functions within the endocytic network (Baust et al., 2008; Baumann et al., 2014; Traikov et al., 2014; Dolat and Spiliotis, 2016; Song et al., 2017).

To initially validate the proteomic data, we turned to quantitative western analysis to probe the presence of huntingtin (Htt) and selected septins (SEPT2, SEPT7 and SEPT9) in a series of GFP-nanotrap immunoprecipitates from RPE-1 cells expressing GFP-tagged versions of the SNX-BAR proteins SNX1 and SNX9, the SNX-FERM-like protein SNX17, and SNX21 (Fig. 3C). This validated the association of these proteins with SNX21 and revealed the specific nature of these interactions, at least within the context of this sorting nexin cohort (Table S6). Finally, immunoprecipitation of endogenous SNX21 confirmed its ability to associate with endogenous Htt (Fig. 3D; Table S7). However, we were unable to reproducibly quantify a robust immunoprecipitation of endogenous SEPT2, SEPT7 and SEPT9 with endogenous SNX21 (data not shown).

### The N-terminal region of SNX21 is required for Htt association

**Fig. 4.**
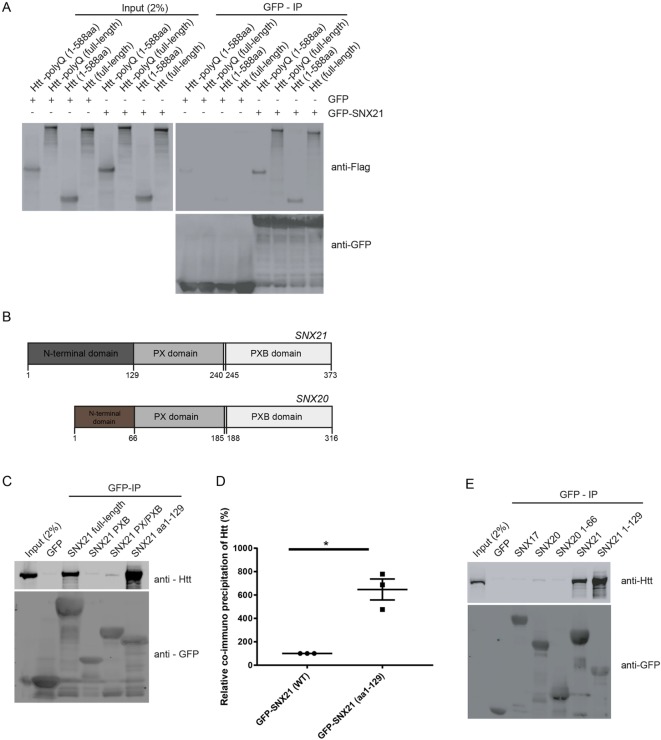
**A putative binding region in the N-terminus of SNX21 facilitates an association with the N-terminal region of both wild-type and mutant (polyQ) huntingtin.** Unless otherwise stated, all interactions were analysed through GFP-nanotrap precipitations from transiently transfected HEK293T cells followed by SDS-PAGE and western blotting. (A) HEK293-T cells expressing GFP-SNX21 or GFP, were co-expressed with either Flag-tagged variants of full-length Htt, the first 588 amino acids of Htt [Htt (1-588aa)], full-length Htt containing a polyQ expansion [Htt-polyQ (full length)] or the corresponding expansion in the first 588 amino acids [Htt-polyQ (1-588aa)]. Each of the huntingtin variants expressed were co-immunoprecipitated by SNX21. Data are representative of three biological replicates. (B) Domain organisation of SNX21 and SNX20. Both proteins have predicted PX and PXB domains, however SNX21 has an extended region N-terminal of the PX domain. (C) The indicated GFP fusions of SNX21 and various truncations were analysed for the ability to precipitate endogenous Htt. The association of Htt was intensified when the N-terminal extension of SNX21 was used as bait. Data are representative of three biological replicates. (D) Fluorescence-based quantitative western blotting was used to access the relative increase in binding to Htt to the SNX21 N-terminus over the wild-type protein (graph represents the mean of three biological replicates and error bars are s.e.m). Significance of change was accessed using unpaired *t*-test and values for Htt were normalised relative to the intensity of the respective precipitated GFP tags. (E) Cells transiently transfected to express GFP-tagged chimeras of full-length SNX21, SNX20 and SNX17, or the N-terminal region of SNX21 (residues 1-129) or SNX20 (residues 1-66) were processed as above. Htt precipitation is highly specific to SNX21 and occurs through the N-terminal extension preceding the PX domain. Data are representative of three biological replicates.

To validate the association of SNX21 with Htt, we co-expressed in RPE-1 cells GFP-SNX21 alongside FLAG-tag chimeras of full-length Htt or a truncated Htt mutant encoding the first 588 amino acids (Fig. 4A). This established that SNX21 associated with the Htt N-terminal region. This region of Htt includes the pathogenic mutations of exon 1 within the *HTT* gene that through expansion of multiple CAG trinucleotide repeats leads to the encoding of a polyglutamine tract (polyQ) (Saudou and Humbert, 2016). Expansion of the polyQ tract beyond 40 or more repeats leads to pathogenicity through mechanisms that are generally considered to arise from perturbed protein-protein interactions leading to toxic gains of function and the possible loss or modification of normal Htt function (Saudou and Humbert, 2016). Inclusion of a 116-residue polyglutamine expansion in either full-length Htt or the N-terminal 1-588 aa region did not adversely affect the ability of SNX21 to bind to Htt (Fig. 4A).

**Fig. 5.**
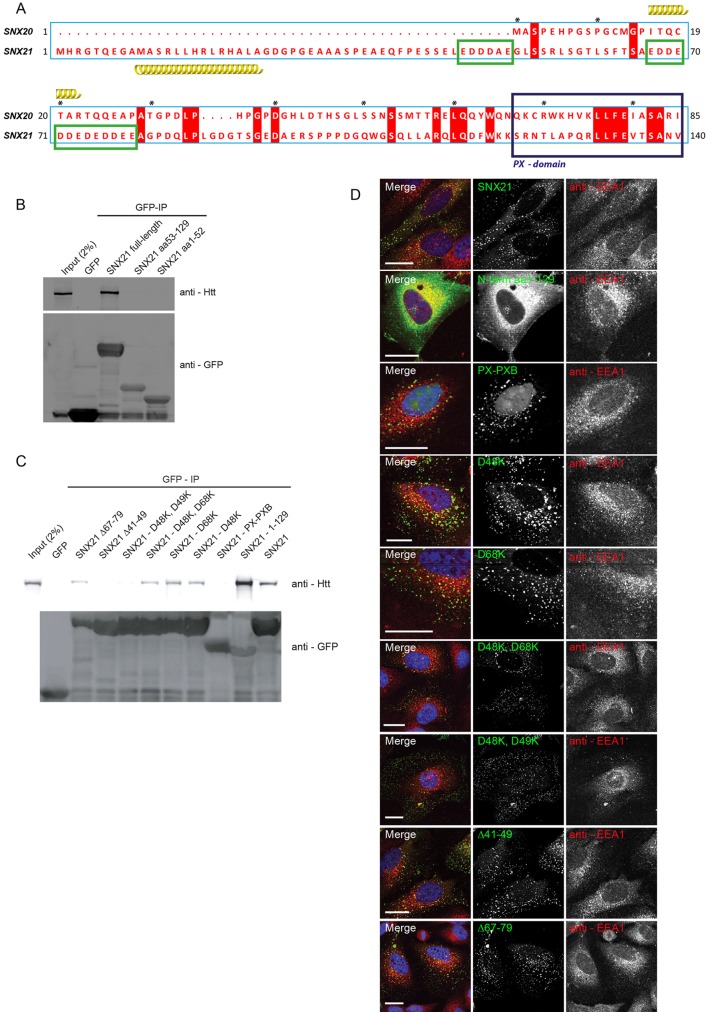
**Co-immunoprecipitation of Htt with SNX21 requires negatively charged residues in the SNX21 N-terminus, but does not require SNX21 to be endosomally localised.** (A) Adaptation of SNX20 and SNX21 protein alignment previously generated by Clairfeuille and colleagues (Clairfeuille et al., 2015)*.* Green boxed regions represent clusters of negatively charged amino acids not present in the SNX20 N-terminal extension. Red boxes denote conserved amino acids within SNX20 and SNX21 sequences and asterisks count every ten residues starting with the fist methionine of SNX20. (B) HEK293-T cells were transiently transfected to express GFP, GFP-tagged full-length SNX21 and two truncation mutants representing the two halves of the N-terminal region of SNX21. Precipitates from the GFP-nanotrap-isolated variants were analysed by western blotting and demonstrate the necessity for a full N-terminal extension in order to facilitate Htt binding. Data are representative of three biological replicates. (C) Site-directed mutagenesis was used to engineer a variety of charge swap mutants targeting the negatively charged clusters of amino acids, prior to probing for Htt binding as above. Two aspartic acid residues in the first N-terminal cluster are essential for precipitation of Htt with SNX21. (D) Both the point mutated GFP-SNX21 and truncation variants were expressed in HeLa cells prior to fixation and immunolabelling with anti-EEA1. Except for the N-terminal 1-129 construct, which lacks a PX domain, all mutants retained an endosomal localisation. Scale bars: 20 µm.

To map the binding site(s) required for SNX21 association with Htt, we first designed a series of truncation mutants based on the known domain architecture of SNX21 (Clairfeuille et al., 2015). Residues 1-129 encode the unique N-terminal domain, while residues 129-240 and 245-372 encode the PX and PXB domains, respectively (Fig. 4B). Transient transfection of HEK-293 cells with plasmids encoding GFP-tagged fusion of each of these regions followed by GFP-nanotrap immunoisolation revealed that although GFP-SNX21 PX (residues 129-240) and GFP-SNX21PX/PXB (residues 129-373) failed to associate with Htt, a fusion encoding the N-terminal domain, GFP-SNX21(1-129) displayed robust Htt binding that was quantitatively (Table S8) more pronounced than that observed with the wild-type GFP-SNX21 (Fig. 4C,D). Further analysis of this region, in particular its sequence relationship to SNX20 (Fig. 4B), revealed that residues 1-53 encode an extension unique to SNX21, while residues 53-129 display a very low level of sequence homology with SNX20 (Fig. 5A). Consistent with this low level of similarity, transient transfection of plasmids encoding GFP-tagged fusions of full-length SNX20 or the N-terminal 1-66 residues of SNX20 immediately preceding the PX domain, established that SNX20 does not associate with Htt (Fig. 4E).

### Acidic clusters in the SNX21 N-terminus are required for Htt association

To further define the binding of the N-terminal region of SNX21 to Htt, we next transiently transfected HEK-293 cells with plasmids encoding GFP-tagged fusions of residues 1-52 and 53-129 of SNX21 followed by GFP-nanotrap immunoisolation. This revealed that neither fusion protein was able to associate with Htt (Fig. 5B).

While studying the 1-129 region of SNX21 further, our attention was drawn to two acidic clusters within residues 41-79 in the N-terminal region of SNX21 (Fig. 5A). As one cluster, ^41^E-S-S-E-L-E-D-D-D-A-E^49^, lay within residues 1-52 and the other, ^67^E-D-D-E-D-D-E-D-E-D-D-E-E^79^, lay within residues 53-129, and as neither of the two clusters was conserved in SNX20, we reasoned that these acidic clusters may constitute an important element in the binding of SNX21 to Htt. To explore this, we derived two GFP-tagged deletion mutants targeting each individual cluster, GFP-SNX21(Δ41-49) and GFP-SNX21(Δ67-79). GFP-nanotrap immunoisolation of corresponding proteins expressed in HEK-293 cells established that both deletions reduced the level of Htt binding with the most pronounced effect being observed with GFP-SNX21(Δ41-49), where a complete loss of binding was observed (Fig. 5C). Further revealing the importance of this acidic cluster, site-directed mutagenesis designed to engineer a double Asp48Lys and Asp49Lys charge switch, GFP-SNX21(D48K, D49K), also led to a complete loss of Htt binding, while reduced binding was observed with the single GFP-SNX21(D48K) mutant (Fig. 5C). For the second acidic cluster, a single GFP-SNX21(D68K) mutant displayed a reduced level of Htt binding, which was further reduced, but not completely lost, when combined with the GFP-SNX21(D48K) mutant to form the GFP-SNX21(D48K, D68K) double mutant (Fig. 5C). Given that the N-terminal 1-129 region of SNX21 is not predicted to contain an overall structural fold apart from an α-helix at residues 10-23 (Clairfeuille et al., 2015), one interpretation of these data is that SNX21 binding to Htt comprises a multivalent association that drives an avidity-based mode of interaction.

Finally, we performed confocal microscopy on all of the deletion and site-directed mutants to examine the relationship between Htt binding and their subcellular distribution. All of the mutants retained their ability to associate with endosomes, establishing that their ability to bind Htt occurs independently of their endosomal association (Fig. 5D). Indeed, the GFP-SNX21(1-129) truncation, which robustly associates with Htt (Fig. 4C,D), is entirely cytosolic, as would be expected given that it lacks the functional PtdIns(3)P- or PtdIns(4,5)P_2_-binding PX domain (Fig. 5D). Together, these data suggest that the association of SNX21 with Htt is mediated through multivalent binding to two acidic clusters present within the unique N-terminal domain of SNX21, of which the N-terminal proximal cluster is the most dominant.

### The PXB domain of SNX21 is required for binding to septins

**Fig. 6.**
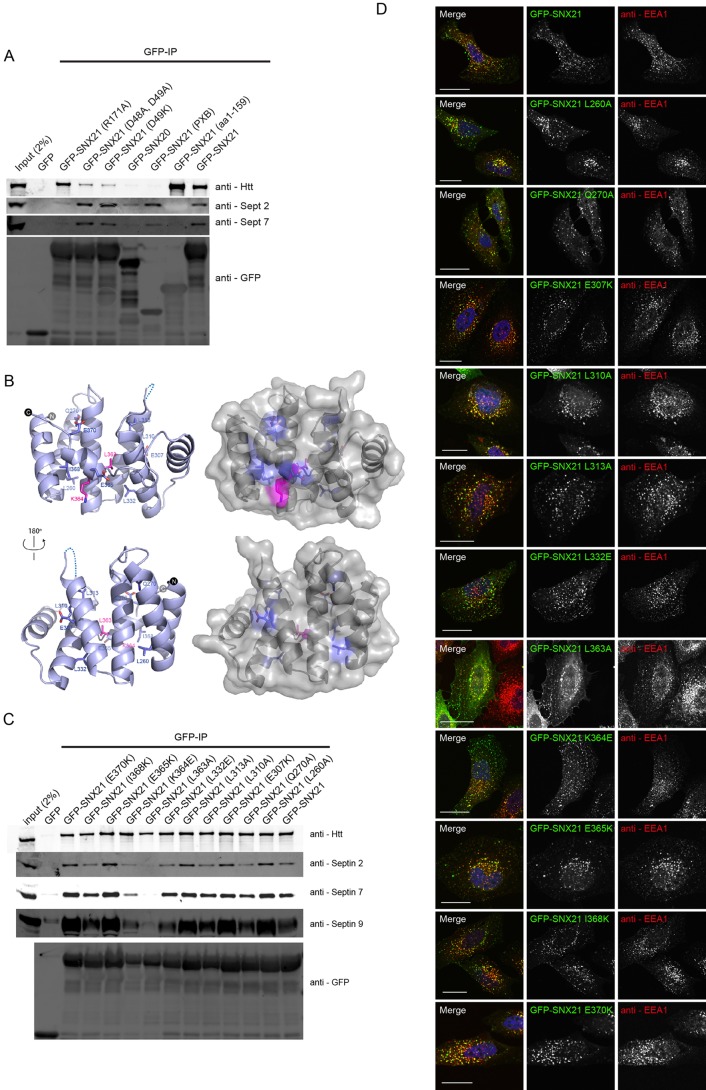
**Co-immunoprecipitation of septins with SNX21 requires a surface exposed leucine in the PXB domain.** (A) HEK293-T cells were transiently transfected with constructs encoding GFP, GFP-SNX20, GFP-SNX21 and various SNX21 point mutants. After GFP-nanotrap immunoisolation, precipitates were analysed by SDS-PAGE and western blotting. GFP-SNX21 precipitates both septin 2 and septin 7, an interaction that occurs via the SNX21 PXB domain and appears to require the endosomal localisation of SNX21. Data are representative of three biological replicates. (B) Amino acid residues mutated in the current study mapped onto the published structure of the mouse SNX21 (Clairfeuille et al., 2015). (C) Site-directed mutagenesis of the SNX21 PXB domain, targeting predicted surface exposed residues. Constructs encoding GFP-tag chimeras of the various SNX21 mutants were transiently expressed in HEK293-T cells prior to GFP-nanotrap, SDS-PAGE and western blotting. Mutation of an evolutionarily conserved leucine (L363A) and a neighbouring lysine (K364E) is sufficient to perturb association with both septin 2 and septin 7. Data are representative of three biological replicates. (D) GFP-SNX21 mutants were expressed in HeLa cells prior to fixation and immunolabelling with anti-EEA1. Each of the mutants analysed retained an endosomal localisation in accordance with the wild-type protein. Scale bars: 20 µm.

In performing an equivalent analysis to map the binding of SNX21 to septins, we observed that those SNX21 site-directed mutants defective in their ability to bind Htt, namely GFP-SNX21(D48K, D49K) and GFP-SNX21(D49K), retained their association with SEPT2 and SEPT7 (Fig. 6A). Further evidence of a distinct mode of binding arose from analysing the SNX21 deletion mutants. While the Htt-binding N-terminal region of SNX21 failed to associate with these septins, the isolated SNX21 PXB domain (residues 273-373) displayed clear binding (Fig. 6A). Consistent with binding being mediated through the PXB domain, a less robust but still apparent association was observed between GFP-tagged SNX20 and septins (Fig. 6A). Interestingly, binding of SNX21 to septins was dependent on the endosomal association, as binding was lost with the GFP-SNX21(R171A) mutant that lacks endosome association (Fig. 6A).

The SNX21 PXB domain consists of TPR α-helical repeats displaying the typical helix-turn-helix motif of TPR proteins (Clairfeuille et al., 2015). The initial six α-helical folds form three TPR repeats, with the seventh and C-terminal α-helical fold looping atypically back across the core TPR structure (Clairfeuille et al., 2015). This unusual conformation leads on the one hand to the exposure of a hydrophilic surface but on the other hand occludes a conserved surface of hydrophobic residues, both surfaces comprising potential sites for protein-protein interactions (Clairfeuille et al., 2015). To further map the binding of septins, we engineered a series of site-directed mutants targeting conserved residues that generally located to the surface-exposed faces of the TPR repeats (Fig. 6B). Within human SNX21, L260 and E270 lie on the α2 helix, E307, L310 and L313 residues on the α4 helix, L332 the α5 helix, and L363, K364, E365, I368 and E370 are all located along the α7 helix. Mutagenesis of these residues, either to the small hydrophobic alanine residue or by introduction of positive and negatively charged amino acids [glutamic acid (E) and lysine (K)], followed by their expression in RPE-1 cells as N-terminal-tagged GFP fusions established that all mutants, after GFP-trap immunoisolation, retained binding to SEPT2, SEPT7 and SEPT9 with the exception of GFP-SNX21(L363A) and GFP-SNX21(K364E) where the level of septin binding was clearly reduced (Fig. 6C). Introduction of these mutations did not globally affect the SNX21 fold as binding to Htt was retained (Fig. 6C) and all mutants localised to intracellular cytosolic punctae, consistent with endosomal association (Fig. 6D). Overall, these data establish that the organisation and/or properties of the α7 helix are an important feature necessary for septin binding.

### SNX21 recruits endogenous Htt on to endosomes

**Fig. 7.**
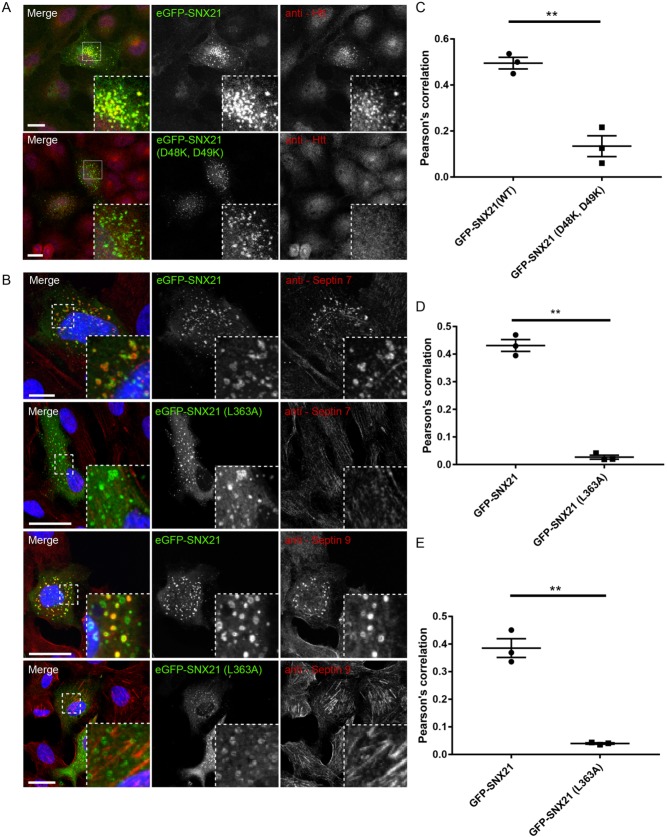
**The endosomal localisation of Htt and septins are perturbed in HeLa cells expressing GFP-SNX21 binding mutants.** (A,B) HeLa cells were transiently transfected with constructs encoding GFP-SNX21 or respective GFP-SNX21 mutants that do not bind to Htt or septins. Cells were fixed and immunostained for endogenous Htt, septin 7 or septin 9. (C-E) Pearson's correlation co-efficients between GFP-SNX21 and endogenous proteins were quantified from digital confocal images using Volocity. GFP-SNX21 (D48K, D49K) demonstrated a quantifiable loss in Pearson's correlation with Htt when compared with the wild-type protein, as was observed for GFP-SNX21 (L363A) with respect to septin 7 and septin 9. Graphs represent the mean of three biological replicates, with ≥7 cells quantified from each. Error bars indicate s.e.m; scale bars: 20 µm. An unpaired *t*-test was used to compare the significance of change with respect to the amount of Htt, septin 7 or septin 9 precipitated with wild-type and mutant SNX21.

At the heart of the proposed scaffolding role of SNX21 is the notion that this sorting nexin serves to recruit and assemble functional protein complexes onto the endosomal membrane (Clairfeuille et al., 2015). In RPE-1 cells, indirect immunofluorescence staining of endogenous Htt revealed a general cytosolic distribution whereas staining of endogenous SEPT7 and SEPT9 displayed the previously documented association with actin stress fibres (Fig. 7A,B) (we were unable to detect endogenous SEPT2 using immunofluorescence in these cells). In contrast, when these cells were transiently transfected to express GFP-SNX21, Htt and the septins showed a steady-state association with the SNX21-positive endosomal population (Fig. 7A,B). Under these conditions, the SNX21-driven endosomal recruitment of Htt was dependent upon binding to SNX21 as expression of the Htt loss-of-binding GFP-SNX21(D48K, D49K) mutant did not elicit the quantitative recruitment of endogenous Htt to those GFP-SNX21(D48K, D49K)-positive endosomes (Fig. 7C; Table S9). In a similar manner, the septin loss-of-binding GFP-SNX21(L363A) mutant failed to drive the quantitative association of SEPT7 or SEPT9 with those GFP-SNX21(L363A)-positive endosomes (Fig. 7D,E; Tables S10, S11). Together, these data establish that under these conditions, the expression of SNX21 leads to a reconfiguration of the steady-state localisation of Htt and septins to endosomes in a manner that is consistent with its proposed role as an endosomal scaffold (Clairfeuille et al., 2015).

## DISCUSSION

The SNX-PXB subfamily of sorting nexins comprises SNX20 and SNX21. Based on the predicted structural properties of their common PXB domains, these proteins have been considered to function as endosome-associated scaffolds for the assembly of protein complexes that regulate the biology of this network of membrane-demarcated compartments (Clairfeuille et al., 2015). However, evidence in support of such a scaffolding role is lacking. Here, we utilised unbiased quantitative proteomics to provide an initial molecular insight into the scaffolding role of the SNX-PXB subfamily (Clairfeuille et al., 2015). Through a focused analysis of SNX21, we have established that its unique N-terminal region is required to associate with Htt while its PXB domain appears to engage members of the septin family of small GTPases. In an *in vitro* cell-culture-based expression system, we present evidence that these individual interactions are required to recruit endogenous Htt and endogenous septins to SNX21-decorated early endosomal populations. With the ever-increasing body of evidence suggesting that septins (Baust et al., 2008; Traikov et al., 2014; Dolat and Spiliotis, 2016) and Htt (Pal et al., 2006; Li et al., 2009; Li et al., 2010; Caviston et al., 2011; Nath et al., 2015) play important functional roles in endosomal biology, our study paves the way for a more-focused analysis of the potential role of SNX21 (and SNX20) in these events.

The major SNX21 interactor characterised in our study is Htt, an interaction that was also recently identified in a large-scale human proteomic analysis (Huttlin et al., 2017). Htt has been implicated in the function of the endo-lysosomal network (Kegel et al., 2005; Pal et al., 2006; Li et al., 2009), in particular through its association with microtubules motors. Htt associates with the minus-end-directed microtubule motor, dynein (Caviston et al., 2007) and through the Htt-associated protein 1 (HAP1) kinesin-1 and the p150^Glued^ subunit of dynactin (Engelender et al., 1997; Li et al., 1998; McGuire et al., 2006; Twelvetrees et al., 2010). These associations facilitate the anterograde and retrograde movement of a variety of organelles that include endosomes and lysosomes (Caviston et al., 2011; Liot et al., 2013), autophagosomes (Wong and Holzbaur, 2014) and vesicles containing SNAREs, APP (amyloid precursor protein), BDNF (brain-derived neurotrophic factor) and GABA receptors (Colin et al., 2008; Gauthier et al., 2004; Her and Goldstein, 2008; Twelvetrees et al., 2010). In addition, Htt engages with Rab GTPases to regulate endosomal function. Htt regulates Rab11 in order to promote vesicle recycling, especially in the context of epithelial polarity (Li et al., 2008; Elias et al., 2015), and forms complexes with Rab5 and Rab8 to modulate actin- versus microtubule-based vesicle motility (Hattula and Peranen, 2000; Sahlender et al., 2005; Pal et al., 2006). Given the importance of microtubule- and actin-based motors in the short- and long-range movement of transport carriers (Granger et al., 2014), our data suggest that the association of SNX21 with Htt may serve to coordinate the directed movement of a specific set of cargo-enriched transport carriers. That SNX21 binding to Htt is neither enhanced nor perturbed by inclusion of a pathogenic polyQ expansion establishes that the interaction reflects a ‘normal’ mode of Htt function rather than a gain or loss of function.

The other SNX21 interactors identified from our proteomic analysis are septins, a family of proteins that share a central GTPase domain through which they hetero-oligomerise to form apolar filaments and higher-ordered assemblies. There are 13 human septins classified into the SEPT2, SEPT3, SEPT6 and SEPT7 subgroups (Kinoshita, 2003). Septins associate with the cytosolic surface of membranes and this may serve to support their filamentous assembly (Bridges et al., 2014). Septin filaments are considered to function by restricting the lateral mobility of integral membrane proteins and to scaffold the assembly of functioning complexes via recruitment of additional protein factors (Caudron and Barral, 2009). In the SEPT2-SEPT6-SEPT7 complex, bending of the filament allows the sensing and induction of positive membrane curvature, that is the remodelling of the membrane towards the cytosol as observed in, for example, membrane tubules (Tanaka-Takiguchi et al., 2009; Bridges et al., 2016). Besides roles in endocytic carrier formation at the cell surface, septins engage a number of elements known to play important roles in the endo-lysosomal network including the AP-3 adaptor, SNARE proteins and the exocyst complex (Hsu et al., 1998; Beites et al., 1999; Taniguchi et al., 2007; Baust et al., 2008; Ito et al., 2009; Traikov et al., 2014; Dolat and Spiliotis, 2016). In describing the association of septins with the early-endosomal-resident SNX21, we have added to this increasing body of evidence reporting a link between septins and the endo-lysosomal network. Our evidence showing that overexpressed SNX21 can recruit endogenous septins to early endosomes, in a manner that is dependent on the membrane association of SNX21 for its septin interaction, hints at a potential role in aiding the formation of endosome-associated septin filaments. That the association is mediated through the organisation and/or properties of the α7 helix is consistent with the recently proposed model for protein-protein interaction within the PXB domain (Clairfeuille et al., 2015). Here, the α7 helix regulates the exposure of a conserved hydrophobic pocket (Clairfeuille et al., 2015). Overall, taken alongside evidence that SNX-PXB proteins can bind to integral membrane cargo proteins (Schaff et al., 2008), we suggest that SNX21 may serve to coordinate the process of cargo recognition via the formation of septin filaments in order to restrict lateral mobility of the captured cargo and aid the biogenesis of cargo-enriched transport carriers.

In sum, our identification of the association of SNX21 with Htt and septins coupled with our initial analysis of these interactions provides a framework from which to further explore the endosomal scaffolding role of the SNX-PXB proteins. Further ongoing experiments are aimed at providing a more detailed functional analysis of SNX21 in endo-lysosomal cargo sorting and the importance of its association with Htt and possibly septins.

## MATERIALS AND METHODS

### Antibodies

Primary antibodies employed in this study were: mouse anti-EEA1 (BD Biosciences #610456, IF 1:200), mouse anti-SNX1 (BD Biosciences #611482, IF 1:200), rabbit anti-VPS35 (Abcam #ab97545, IF 1:400), sheep anti-TGN38/46 (Genetex #GTX74290, IF 1:400), mouse anti-CD63 (Santa Cruz #MEM259, IF 1:250), mouse anti-LAMP1 (Hybridoma Bank #H4A3, IF 1:400), rabbit anti-Huntingtin (Abcam #ab109115, IF 1:400 and WB 1:1000), mouse anti-Huntingtin (Thermo Fisher 3-19, IF 1:200) rabbit anti-Septin 2 (Abcam #ab179436, WB 1:1000), rabbit anti-Septin 7 (Proteintech #13818, IF 1:200 and WB 1:1000), rabbit anti-Septin 9 (Proteintech # 10769, IF 1:200 and WB 1:1000), mouse anti-GFP (Roche #11 814 460 001, WB 1:2000), rabbit anti-SNX21 (Proteintech #22193-1-AP, WB 1:1000), mouse anti-rabbit conformation specific (Cell Signaling #3678, WB 1:1000) and rabbit IgG (EPR25A) isotype control (Abcam #172730). Jackson ImmunoResearch Alexa-Fluor680/800-conjugated secondary antibodies were used for quantitative detection of proteins by western blot at 1:18,000 and Invitrogen Alexa-Fluor secondary antibodies for detection of primary antibodies in immunofluorescence experiments at 1:400.

### Cell culture

HeLa, HEK293-T and RPE1 cells were cultured at 37°C and 5% CO_2_ in DMEM supplemented with 10% FCS and 1% penicillin-streptomycin. For SILAC experiments, RPE1 cells were cultured with labelled amino acids (R6K4 or R0K0) contained in SILAC-DMEM (Sigma) supplemented with 10% dialysed FCS (Sigma).

### Viral and transient expression of tagged constructs

RNA was isolated from HeLa cells using the RNeasy (Qiagen) kit prior to cDNA generation using QuantiTect Reverse Transcription Kit (Qiagen) both in accordance with manufacturer's instructions. Using primers designed against the wild-type SNX21 sequence, the ORF of SNX21 was cloned into pEGFP-C1 (Clonetech). Subsequently, eGFP-C1-SNX21 was used as template to generate point mutations into the wild-type ORF by site-directed mutagenesis using the Agilent Quik-Change Kit, oligonucleotide mutagenic primers (see Table S1) both contained the desired mutation and were designed to anneal at overlapping sequences on the opposing strands. SNX21 was divided into its sub-domains in accordance with the boundaries defined by Clairfeuille and colleagues (Clairfeuille et al., 2015)*.* From pEGFP-C1 we sub-cloned the ORF of SNX21 into the lentiviral expression plasmid pXLG3; HEK293-T cells were then transiently transfected with pXLG3-GFP-SNX21 in combination with viral helper plasmids and viral medium harvested after 48 h growth. HeLa or RPE1 cells were exposed to decreasing titres of lentivirus for 72 h and virally transduced cells expressing low levels of eGFP-SNX21 were selected for subsequent experimentation. All other SNX-containing plasmids were generated in the lab previously and were also expressed from the eGFP-C1 vector. Full-length huntingtin variants were cloned into pTRE2hyg-FLAG and DNA encoding Huntingtin 1-588 into pC1-FLAG, both of which were kind gifts from Professor David Rubinstein (Cambridge Institute for Medical Research, Cambridge, U.K.). For transient expression in HEK-293-T, we cultured cells in Optimem containing plasmid DNA with polyethylenimine (PEI) at a 3:1 ratio (3 µg DNA:1 µg PEI) for 4 h prior and processed after growth for a further 24 h. Transient transfection of plasmid DNA in HeLa and RPE1 cells was performed using Lipofectamine LTX according to the manufacturer's protocol.

### SILAC experiments

SILAC reagents were purchased from Thermo Fisher with the exception of FCS and SILAC DMEM from Sigma. RPE1 cells virally expressing selected plasmids were seeded in six-well plates in SILAC labelling medium supplemented with dialysed FCS and cultured over a minimum of six passages to achieve full labelling with respective isotopes and a minimum of two confluent 20 cm dishes for generation of lysates. GFP-expressing control cells were grown in unlabelled medium with standard arginine and lysine (R0K0) and GFP-SNX21-expressing cells were grown in medium supplemented with ‘medium’-mass isotopes [^13^C_6_]-arginine and 4,4,5,5-D4-Lysine (R6K4). Cells were lysed in immunoprecipitation buffer [50 mM Tris-HCl (pH 7.4), 0.5% NP40, 1 mM PMSF, 200 µM Na_3_VO_4_ and a Roche mini complete protease inhibitor tablet] and the GFP tags were precipitated with GFP-nanotrap beads (Chromotek) for 1 h at 4°C then combined prior to three washes in wash buffer (50 mM Tris-HCl, pH7.4, 0.2% NP40). Proteins were isolated in sample buffer, separated using NuPAGE (4-12%) pre cast gels (Invitrogen), visualised using All Blue protein stain (Invitrogen) and analysed by LC-MS-MS on an Orbitrap Velos (Thermo) spectrophotometer (Steinberg et al., 2012; Steinberg et al., 2013; McGough et al., 2014a,b; McMillan et al., 2016).

### Immunoprecipitation experiments

For GFP-nanotrap, HEK293-T cells were transfected with respective GFP constructs as described above, lysed in immunoprecipitation buffer (50 mM Tris-HCl, pH 7.4, 0.5% NP40, 1 mM PMSF, 200 µM Na_3_VO_4_ and a Roche mini complete protease inhibitor tablet) and the GFP tags were precipitated with GFP-nanotrap beads (Chromotek) for 1 h at 4°C, washed three times in wash buffer (50 mM Tris-HCl, pH 7.4, 0.2% NP40), prior to separation by SDS-PAGE and western blotting. For the endogenous immunoprecipitations; HEK293-T cells were lysed in immunoprecipitation buffer (50 mM Tris-HCL, pH 7.4, 0.5% NP40, 1 mM PMSF, 200 µM Na_3_VO_4_ and a Roche mini complete protease inhibitor tablet). Cleared lysates were separated into two fractions prior to the addition of 2 µg of either anti-SNX21 or rabbit IgG control antibody and incubated at 4°C for 2 h. Protein-G agarose beads (Pierce) were then washed three times in lysis buffer and added to the respective lysates prior to a further incubation at 4°C for 1 h. Beads were washed three times in wash buffer (50 mM Tris-HCl, pH 7.4, 0.2% NP40), prior to separation by SDS-PAGE and western blotting.

### Western blotting

Western blots were performed using standard procedures; proteins were detected and quantified on a LI-COR Odyssey infrared scanning system via recognition of fluorescently labelled secondary antibodies.

### Image acquisition and quantification

HeLa or RPE1 cells virally transduced to express low levels of GFP-SNX21 (co-localisation assays) or transiently transfected with plasmid DNA (Htt and septin endosomal recruitment assays) were plated on glass coverslips, fixed with 4% (w/v) PFA and permeabilised using 0.1% Triton X-100 (Sigma) for 4 min, except for visualisation of septins, where cells were fixed and permeabilised in ice-cold methanol for 4 min at 4°C. Cells were blocked in PBS supplemented with 2% BSA (P-BSA) for 20 min at room temperature, incubated on droplets of 0.2% P-BSA containing primary antibody for 1 h, washed in PBS, prior to incubation on droplets of 0.2% P-BSA containing Alexa Fluor secondary antibodies (Invitrogen) and DAPI reagent. All cells were imaged using a Leica SP5 confocal laser-scanning microscope with a 63×1.40-0.60 PL Apo λBL oil immersion objective. For quantitative co-localisation measurements, we performed the same analyses as described previously (van Weering et al., 2012a).

### Statistical analysis

For quantification of western blots, all data represent the mean of three independent experiments, which in each case provided adequate power to determine the statistical significance of any changes observed. The raw data from the quantitative western blotting was normalised relative to the level of the respective precipitated GFP tag. For all analyses the mean and standard error were calculated followed by a two-tailed, unpaired *t*-test for determination of statistical significance and *P*<0.05 was determined as significant. Data points for all quantified experimentation and statistical analyses are included in Tables S4-S11. All graphs were generated using PRISM-GraphPad. Quantitative co-localisation analyses were performed using Volocity; a minimum of 22 cells were analysed for comparison of localisation with endogenous markers and a minimum of 15 cells were used for Rab co-expression studies.

## Supplementary Material

Supplementary information
