# Supplementary material for: Sorting nexin-21 is a scaffold for the endosomal recruitment of huntingtin
Source: J Cell Sci. 2018 Sep 10;131(17):jcs211672. doi: 10.1242/jcs.211672 (PMC6140323; doi:10.1242/jcs.211672)
Supplement: Supplementary information [file joces-131-211672-s1.pdf]

Supplementary Table 1 – Primer sequences used for site-directed mutagenesis.

Mutation		***Primer Sequence (5'-3')***
D48K	(sense)	ggccctcggcgtccttgtcctccagctct
	(antisense)	agagctggaggacaaggacgccgagggcc
D68K	(sense)	cgtcgtcctcgtccttctcggcgctggtg
	(antisense)	caccagcgccgagaaggacgaggacgacg
D48K, D49K	(sense)	ctcagagctggaggacaagaaggccgagggcctgtcct
	(antisense)	aggacaggccctcggccttcttgtcctccagctctgag
D68K,D71K,D74K,D77K	(sense)	ccagcctcctccttgtcctccttctcgtccttctcgtccttctcggcgctgg
	(antisense)	ccagcgccgagaaggacgagaaggacgagaaggaggacaaggaggaggctgg
Δ41-49	(sense)	acaggccctcggccggaaactgctcg
	(antisense)	cgagcagtttccggccgagggcctgt
Δ67-69	(sense)	ggtcagggccagcggcgctggtgaag
	(antisense)	cttcaccagcgccgctggccctgacc
L260A	(sense)	ccagagtgccgcagcctcacgatagaggcca
	(antisense)	tggcctctatcgtgaggctgcggcactctgg
Q270A	(sense)	gcccagctgggctgccagctgccaggca
	(antisense)	tgcctggcagctggcagcccagctgggc
E307K	(sense)	gggccttcttacagcatgcccgggcct
	(antisense)	aggcccgggcatgctgtaagaaggccc
L310A	(sense)	ccccaagcagctgcgcggccttctcacagc
	(antisense)	gctgtgagaaggccgcgcagctgcttgggg
L313A	(sense)	gctcttgtccccagccagctgcagggcc
	(antisense)	ggccctgcagctggctggggacaagagc
L332E	(sense)	ccaggcgccaggactcccggacatgggcc
	(antisense)	ggcccatgtccgggagtcctggcgcctgg
L363E	(sense)	ttgatgagcaattctttggcactggggggtggtgtgg
	(antisense)	ccacaccaccccccagtgccaaagaattgctcatcaa
K364E	(sense)	tccttgatgagcaattcctcgagactggggggtggtg
	(antisense)	caccaccccccagtctcgaggaattgctcatcaagga
E365K	(sense)	ctccttgatgagcaattttttgagactggggggtg
	(antisense)	caccccccagtctcaaaaaattgctcatcaaggag
I368K	(sense)	ccttagtccagcacctccttctcgagcaattctttgagactgg
	(antisense)	ccagtctcaaagaattgctcgagaaggaggtgctggactaagg
E370K	(sense)	gtaccttagtccagcaccttcttgatgagcaattctttg
	(antisense)	caaagaattgctcatcaagaaggtgctggactaaggtac

Journal of Cell Science • Supplementary information

Supplementary Table 2 - Unfiltered SNX21 interactome obtained from RPE-1 cells

GFP and GFP-SNX21 expressing RPE-1 cells were respectively cultured in light (R0K0) and medium (R6K4) media for at least six doubling to ensure steady-state protein labelling. Thereafter, immuno-isolation of the GFP tag was achieved through GFP-nanotrap prior to mixing of the two samples and protein resolution on SDS/PAGE and protein identification by LC-MS/MS. The unfiltered data shows the presence of around 4,400 proteins. Parameter definitions are:

Accession: Uniprot accession number of the protein

Coverage: Percentage of protein sequence covered by identified peptides

Peptide Spectrum Matchs (PSMs): Total number of peptides identified (including multiples of same peptide sequence)

Peptides: Total number of unique peptides identified (multiples of same peptide sequence count as single peptide)

AAs: Number of amino acids in the protein

[kDa]: Mass of the protein

calc. pI: Calculated isoelectric point of the protein

Score: Combines several parameters; a high score generally indicates high protein abundance and a high confidence of the software in the detection and quantification.

Medium/Light: Ratio of the quantification values of the medium (GFP-SNX21) and light (GFP) quantification channels

Medium/Light Count: Number of peptides quantified used to calculate Medium/Light ratio

Medium/Light Variability (%): Variability of the protein ratios used to calculate the Medium/Light ratio

Description: Name of the protein

Click here to Download Table S2

Supplementary Table 3 - Filtered SNX21 interactome

The raw unfiltered data from Supplementary Table 2 was subjected to filtration based on two criteria: more than 2 peptides detected for an individual protein, and a Medium/Light enrichment ratio (i.e. GFP-SNX21/GFP) of greater than 20. This defined that the SNX21 interactome in RPE-1 cells is comprised of 287 proteins. Parameter definitions are as in the legend to Supplementary Table 2

Click here to Download Table S3

Supplementary Table 4

Raw data; Quantitative analysis of GFP-SNX21 co-localisation (Pearson's correlation) with endogenous markers of the endosomal network.

Click here to Download Table S4

Supplementary Table 5

Raw data; Quantitative analysis of GFP-SNX21 co-localisation (Pearson's correlation) with mCherry-tagged Rab proteins.

Click here to Download Table S5

Supplementary Table 6

Raw data; Quantitative analysis of co-localisation (Pearson's correlation) between GFP-tagged Sorting Nexins and endogenous Huntingtin, Septin 2, 7 and 9.

Click here to Download Table S6

Supplementary Table 7

Raw data; Quantitative analysis of Huntingtin co-immunoprecipitation with endogenous SNX21.

Click here to Download Table S7

Supplementary Table 8

Raw data; Quantitative analysis of endogenous Huntingtin co-immunoprecipitation with GFP-SNX21 (WT) and GFP-SNX21 (aa1-129).

Click here to Download Table S8

Supplementary Table 9

Raw data; Quantitative analysis of endogenous Huntingtin co-localisation with GFP-SNX21 (WT) and GFP-SNX21 (D48K, D49K).

Click here to Download Table S9

Supplementary Table 10

Raw data; Quantitative analysis of endogenous Septin 7 co-localisation with GFP-SNX21 (WT) and GFP-SNX21 (L363A)

Click here to Download Table S10

Supplementary Table 11

Raw data; Quantitative analysis of endogenous Septin 9 co-localisation with GFP-SNX21 (WT) and GFP-SNX21 (L363A)

Click here to Download Table S11

Journal of Cell Science • Supplementary information
